# Evaluation of Automated Disk Diffusion Antimicrobial Susceptibility Testing Using Radian® In-Line Carousel

**DOI:** 10.1007/s00284-024-03710-z

**Published:** 2024-05-30

**Authors:** Kim Callebaut, Anke Stoefs, Kristof Emmerechts, Kristof Vandoorslaer, Ingrid Wybo, Deborah De Geyter, Thomas Demuyser, Denis Piérard, Astrid Muyldermans

**Affiliations:** 1 Department of Microbiology and Infection Control, Vrije Universiteit Brussel (VUB), Universiteit Ziekenhuis Brussel (UZ Brussel), Laarbeeklaan 101, 1090 Brussels, Belgium; 2 AIMS Lab, Center for Neurosciences, Faculty of Medicine and Pharmacy, Vrije Universiteit Brussel (VUB), Laarbeeklaan 103, 1090 Brussels, Belgium

## Abstract

Antimicrobial susceptibility testing (AST) by disk diffusion provides an accurate image of bacterial growth, enabling the detection of culture purity, heterogeneous growth, and antibiotic interactions. However, this manual method is time-consuming and visual interpretation is prone to errors. To overcome these disadvantages, the Radian® In-Line Carousel (Copan, Brescia, Italy) was launched, which is a WASPLab® module dedicated to full automation of (pre)-analytical steps as well as interpretation of disk diffusion AST. However, until now, no evaluation of Radian® against manual disk diffusion has been performed. We assessed the categorical agreement (CA) between standardized disk diffusion (reference method) and Radian® using EUCAST 2021 breakpoints. We tested 135 non-duplicate strains, selected from the National EUCAST challenge panel, clinical strains, and external quality controls. The strains included Enterobacterales (n = 63), *Enterococcus faecalis* (n = 3), *Enterococcus faecium* (n = 10), *Pseudomonas aeruginosa* (n = 16), *Staphylococcus aureus* (n = 19), coagulase-negative staphylococci (n = 4), and *Streptococcus* spp. (n = 20). Furthermore, we explored antibiotic disk thermolability in the WASP Radian® carousel by testing 10 ATCC® strains up to 7 days. The observed CA was 95.3%, 96.3%, 93.8%, 97.3% and 98.0% for Enterobacterales, *Enterococcus* spp., *P. aeruginosa*, *Staphylococcus* spp. and *Streptococcus* spp., respectively, resulting in an acceptable overall CA for all groups. (Very) major error rates were ≤ 5% for all antibiotics. Antibiotic disk thermostability was confirmed up to 4 days in the WASP Radian® In-Line Carousel. The Radian® In-Line Carousel provides a fully automated solution for accurate disk diffusion AST, reducing workload and improving standardization and traceability. In addition, our study demonstrated the thermostability of antibiotic disks up to 4 days in the WASP Radian® In-Line Carousel.

**Supplementary Information:**

The online version contains supplementary material available at 10.1007/s00284-024-03710-z.

## Introduction

Identifying the causative bacteria of an infection and performing antimicrobial susceptibility testing (AST) to determine a susceptibility profile are crucial assignments in clinical microbiology laboratories. The AST profile enables good antibiotic prescribing, optimal patient care, and prevention of bacterial resistance by initiating appropriate escalation or de-escalation of antibiotics [1–3]. Different AST methods utilize the presence or absence of bacterial growth on a solid agar plate or liquid growth medium containing antibiotics to determine the sensitivity of the bacteria [4, 5]. Disk diffusion testing, a commonly used AST method in routine clinical microbiology laboratories, provides a true in vitro image of bacterial growth, enabling the detection of culture purity, heterogeneous growth, and antibiotic interactions [5–7]. The European Committee on Antimicrobial Susceptibility Testing (EUCAST) developed a standardized AST method based on disk diffusion with zone diameter breakpoints correlated with clinical minimal inhibitory concentration (MIC) breakpoints [8]. Disk diffusion is a time-consuming method that requires 16–20 h of incubation to measure inhibition zones. Semi-automated reading of zone diameters can be conducted using SIRScan® (i2a, Montpellier, France), but is still prone to interpretation errors [3, 9].

Designing and implementing laboratory automation systems plays a crucial role in improving productivity, traceability, and quality and is an ongoing process [2, 7, 10]. One such automation system is WASPLab® (Copan, Brescia, Italy), which enables automated inoculation of specimens, incubation of solid plates, and analysis of bacterial cultures through images [11, 12]. Additionally, Copan has developed the Radian® in-line Carousel and Radian® expert System to complement WASPLab® by automating antimicrobial disk diffusion testing and interpretation [2, 3]. It contains a carousel that is capable of holding up to 50 antimicrobial discs. Laboratories are able to customize the combination and location of antimicrobial discs that are placed on the prepared Mueller Hinton plates. In combination with WASPLab® the prepared agars are automatically transferred to the incubators. Following the predefined incubation periods, plate imaging occurs automatically. These images are then processed by the Radian® expert system which performs zone measurement and interpretation.

According to EUCAST and manufacturers’ guidelines, antibiotic disks should be stored in sealed containers, protected from humidity and light, at a temperature lower than 8 °C when not in use. Before use, the antibiotic disks should reach room temperature, and the sealed container should not be opened before this temperature is reached to avoid humidity [6]. Radian® is not equipped with a cooling device, so placing the antibiotic disks at room temperature for long periods when being stored inside. Due to the practical disadvantage of needing to retract up to 50 antibiotic disks from Radian® at the end of each working day and placing them in the fridge, a thermolability study was performed.

We evaluated the automation of disk diffusion AST using WASPLab® with Radian® In-Line Carousel (Copan) against manual disk diffusion with semi-automated reading by SIRScan® (i2a).

## Materials and Methods

### Strains

#### Antimicrobial Susceptibility Testing

135 non-duplicate strains were tested, selected from the National EUCAST challenge panel (n = 28) [13], clinical strains (n = 37) and external quality controls (n = 70) conserved at −80 °C: Enterobacterales n = 63, *Enterococcus faecalis* n = 3, *Enterococcus faecium* n = 10, *Pseudomonas aeruginosa* n = 16, *Staphylococcus aureus* n = 19, coagulase negative staphylococci n = 4 and *Streptococcus* spp. n = 20 (*Streptococcus pneumoniae* n = 10). Different resistance mechanisms were included in this study: 13 extended-spectrum β-lactamase (ESBL)—producing Enterobacterales (*Escherichia coli* n = 7, *Klebsiella oxytoca* n = 1, *Klebsiella pneumoniae* n = 5), 9 carbapenemase producing Enterobacterales (CPE) (VIM-1 producing *Enterobacter cloacae complex* n = 1 and *K. pneumoniae* n = 1, OXA-48 producing *Escherichia coli* n = 1 and *K. pneumoniae* n = 4, KPC producing *K. pneumoniae* n = 2), 3 metallo-beta-lactamase (MBL) producing *P. aeruginosa* (VIM-2 n = 2), 10 vancomycin-resistant enterococci (VRE) (*VanB* positive *E. faecalis* n = 1, *VanA* positive *E. faecium* n = 4, *VanB* positive *E. faecium* n = 4 and one *E. faecium* with unknown *Van* genes) and 15 methicillin-resistant *S. aureus* (MRSA).

#### Thermolability of Antibiotic Disks

10 reference strains, conserved at −80 °C, were tested: *E. coli* ATCC® 25922™ and ATCC® 35218™, *P. aeruginosa* ATCC® 27853™, *K. pneumoniae* ATCC® 700603™, *S. aureus* ATCC® 29213™ and NCTC® 12493™, *E. faecalis* ATCC® 29212™ and ATCC® 51299™, *S. pneumoniae* ATCC® 49619™ and *Campylobacter jejuni* ATCC® 33560™.

### AST Methodology

All strains were processed in parallel in two ways: (i) manually according to EUCAST [8] and (ii) using WASPLab® and Radian® in-Line Carousel. Strains were cultured manually on a non-selective blood agar (Thermo Fisher Scientific, Waltham, USA). These agar plates were incubated for 16 h in a CO_2_ incubator (Thermo Fisher Scientific). Species were identified by MALDI Biotyper® Sirius system (Bruker Daltonics, Bremen, Germany).

#### EUCAST Standardized Disk Diffusion Testing

Standardized disk diffusion testing was performed according to EUCAST methodology [8]. A 0.5 McFardland (McF) solution was made starting from freshly grown colonies in 0.9% saline solution. Four drops of this inoculum were transferred to a 120 mm square Mueller–Hinton (MH) agar (Axonlab, Hengersberg, Germany) and streaking was done manually followed by the addition of 16 antibiotic disks (i2a) with a disk dispenser. For *S. pneumoniae* MH-F agars (Thermo Fisher Scientific) were used as recommended by EUCAST. The MH(-F) agars were incubated for 18 ± 2 h, aerobic for MH-agars and CO_2_-enriched for MH-F agars (Thermo Fisher Scientific). Inhibition zones were read using SIRScan 2000 (i2a) [14]. The inhibition zones were manually adjusted when necessary.

#### WASPLab®/Radian® In-Line Carousel

AST by WASPLab® and Radian® In-Line Carousel was performed according to EUCAST guidelines for standardized disk diffusion adapted for inoculation by WASP® corresponding with manufacturer’s instructions [15]. A 0.5 McF solution was made starting from freshly grown colonies in 0.9% saline solution. A 1/3 dilution of this suspension in phosphate-buffered saline was made, which was further used as inoculum. 60 µL of this inoculum was applied by WASP® to three, 90 mm circular MH agars (Thermo Fisher Scientific). Using the Radian® In-Line Carousel, six antibiotic disks (Thermo Fisher Scientific) were dispensed to each MH-agar for the Gram-negative strains. For the Gram-positive strains, six antibiotic disks were dispensed on two MH-agars and four on a third MH-agar. For *S. pneumoniae* MH-F agars (Thermo Fisher Scientific) were used as recommended by EUCAST. MH(-F) agars were incubated for 16 h in WASPLab® automated incubators (aerobic for MH-agars and CO_2_-enriched for MH-F agars). Subsequently, images were acquired, and inhibition zones were read using WASPLab® Webapp [15]. The inhibition zones were manually adjusted when necessary.

### Antimicrobials

Two antibiotic panels were used, the first panel targets Gram-negative (GN) bacteria and the second panel targets Gram-positive (GP) bacteria.

#### Gram-Negative Bacteria

The GN antibiotic panel contained 18 different antibiotics: ampicillin, amoxicillin-clavulanic acid, piperacillin-tazobactam, temocillin, cefadroxil, cefuroxime, ceftriaxone, ceftazidime, cefepime, meropenem, moxifloxacin, ciprofloxacin, amikacin, gentamicin, aztreonam, fosfomycin, nitrofurantoin, sulfamethoxazole/trimethoprim.

#### Gram-Positive Bacteria

The GP antibiotic panel contained 16 different antibiotics: penicillin, oxacillin, ampicillin, cefoxitin, erythromycin, clindamycin, tetracycline, gentamicin (10 µg and 30 µg), vancomycin, nitrofurantoin, linezolid, rifampicin, moxifloxacin, ciprofloxacin and sulfamethoxazole/trimethoprim.

### Thermolability

According to the manufacturer’s instructions, i2a discs should be stored in a dry place between 2 and 25 °C for a maximum of 35 days, except for specific antimicrobial disks, such as frequently tested beta-lactam antibiotics, which have a stability of only 7 days. When placed in a dispenser, a desiccant must be added, a step not feasible with the Radian® carrousel. For Thermo Fisher scientific discs, once opened, cartridges should be stored in a container provided with a desiccant and at 2–8 °C. The stability after opening under the described conditions is specified on the carton for each antimicrobial disc and is maximum 7 days.

Thermolability testing was performed for 10 reference strains at 7 consecutive days leaving the disks in the Radian In-Line Carousel at room temperature [6, 16]. AST was performed as described above with Wasplab/Radian® In-Line Carousel using MH agars except for *S. pneumoniae* and *C. jejuni* where MH-F agars (Thermo Fisher Scientific) were used. All strains (Microdiagnostics, United States of America; LGC, United Kingdom; DSMZ, Germany; NCTC, United Kingdom; LMG, Belgium) were stored at −80 °C for 5–20 years and were cultured manually on a non-selective blood agar (Thermo Fisher Scientific, Waltham, USA). These agar plates were incubated for 16 ± 2 h in a CO_2_ incubator (Thermo Fisher Scientific).

Antibiotic disks (Thermo Fisher Scientific) were added to each MH(-F) agar based on the identification of the ATCC® strain according to EUCAST guidelines. Agars were incubated for 16 h in the WASP® automated incubator, capable of incubating the MH-F agars in CO_2_ enriched environment. For *C. jejuni* plates were incubated outside the WASP® in a micro-aerophilic environment (Thermo Fisher Scientific). Inhibition zones were read using WASPLab® Webapp, with manual adjustments where necessary [14, 15]. Inhibition zones were evaluated using EUCAST acceptance limits, and potential trends were assessed [16]. This experiment was repeated five times for each strain.

### Quality Control

Quality control strains were performed during each run, utilizing the strains recommended for routine quality control by EUCAST, as outlined in their guidance document version 11, January 2021 [17]. All quality control strains were stored at −80 °C for 5–20 years and were cultured manually on a non-selective blood agar (Thermo Fisher Scientific, Waltham, USA). These agar plates were incubated for 16 h in a CO_2_ incubator (Thermo Fisher Scientific).

### Statistical Analysis

Statistical analyses were performed using Microsoft Excel (version 2016, USA). Categorical agreement (CA) was assessed between standardized disk diffusion and Radian® In-Line Carousel using EUCAST 2021 breakpoints.

## Results

### Antimicrobial Susceptibility Testing

Results of WASPLab® coupled to Radian® In-Line Carousel compared to EUCAST standardized disk diffusion testing are displayed in Table 1.

**Table 1:** Results of AST disk diffusion testing using Radian® In-Line carousel as compared to standardized AST

Group	# isolates	CA (%)	mD (#)	MD (#)	VMD (#)
Enterobacterales (n = 63)					
Amikacin	63	100			
Amoxicillin-clavulanic acid	58	100			
Ampicillin	63	98.4			1
Aztreonam	63	88.9	7		
Cefadroxil	63	100			
Cefepime	63	82.5	11		
Ceftazidime	63	88.9	7		
Ceftriaxone	63	96.8	1	1	
Cefuroxime	45	100			
Ciprofloxacin	59	98.3	1		
Cefotaxime	23	100			
Gentamicin	63	93.6	4		
Meropenem	63	90.5	6		
Moxifloxacin	63	96.8		2	
Nitrofurantoin	27	100			
Piperacillin-tazobactam	61	90.2	6		
Temocillin	44	97.7	2		
Trimethoprim-sulfamethoxazole	63	100			
*Pseudomonas aeruginosa* (n = 16)					
Amikacin	16	100			
Aztreonam	16	87.5	2		
Cefepime	16	93.8	1		
Ceftazidime	16	93.8	1		
Ciprofloxacin	16	100			
Meropenem	16	93.8	1		
Piperacillin-tazobactam	16	87.5	2		
*Enterococcus* spp. (n = 13)					
Ampicillin	13	100			
Ciprofloxacin	13	100			
Gentamicin	13	100			
Linezolid	13	100			
Nitrofurantoin	4	100			
Trimethoprim-sulfamethoxazole	12	92.3	1		
Vancomycin	12	92.3		1	
*Staphylococcus* spp. (n = 23)					
Cefoxitin	23	100			
Ciprofloxacin	23	82.6	4		
Clindamycin	23	100			
Erythromycin	23	100			
Gentamicin	23	100			
Linezolid	23	100			
Moxifloxacin	23	95.6		1	
Oxacillin	19	89.5	2		
Penicillin G	21	100			
Rifampicin	23	100			
Tetracycline	23	100			
Trimethoprim-sulfamethoxazole	23	95.6	1		
Vancomycin	23	100			
*Streptococcus* spp. (n = 20)					
Ampicillin	10	80	2		
Ciprofloxacin	10	100			
Clindamycin	20	100			
Erythromycin	20	95	1		
Linezolid	20	100			
Moxifloxacin	20	100			
Nitrofurantoin	6	100			
Oxacillin	5	100			
Penicillin G	10	100			
Rifampicin	20	95	1		
Tetracycline	20	100			
Trimethoprim-sulfamethoxazole	20	100			
Vancomycin	20	100			

*CA* categorical agreement, *mD* minor discrepancy, *MD* major discrepancy, *VMD* very major discrepancy

#### Enterobacterales

Overall CA of 95.2% was obtained among the 63 Enterobacterales strains that were tested. Within the discrepancies, 4.4% were mD, three were major discrepancies (MD) and one was a very major discrepancy (VMD). Disk diffusion screening cut-off for CPE, according to EUCAST methodology (meropenem inhibition zone diameter < 25 mm), enabled the detection of all CPE isolates. In addition, ESBL screening method according to EUCAST methodology (cefotaxime 5 µg inhibition zone < 21 mm OR ceftriaxone 30 µg inhibition zone < 23 mm AND ceftazidime inhibition zone < 22 mm) allowed detection of 11 ESBL producing isolates using SIRScan® and 12 ESBL producing isolates using Radian® coupled to WASPLab®.

#### *Pseudomonas aeruginosa*

An overall CA of 93.8% between both methods was obtained for the 16 *P. aeruginosa* isolates, with all discrepant results being mD. Disk diffusion screening cut-off for CPE, according to EUCAST methodology (meropenem inhibition zone diameter < 25 mm), enabled the detection of all three MBL producing *P. aeruginosa* strains.

#### *Enterococcus* spp.

Among the 13 *Enterococcus* spp. isolates a CA of 96.4% was obtained between both methods with 1.2% mD. One MD was observed in a *VanB* + *E. faecalis* isolate for vancomycin. However, this appeared to be a correct identification of the VRE strain by Radian® Coupled to WASPLab and a false susceptibility for vancomycin on SIRScan® (external quality control strain). All other VRE isolates were categorized correctly using both methods.

#### *Staphylococcus* spp.

Overall CA of 97.3% was obtained with 2.4% mD and one MD. All MRSA isolates were categorized correctly using EUCAST methodology (cefoxitin 30 µg inhibition zone diameter < 22 mm).

#### *Streptococcus *spp*.*

Overall CA of 98.0% was obtained with all discrepant results being mD.

### Thermolability of Antimicrobial Disks

Table 2 displays the minimum and maximum amount of days for which the antibiotic disk inhibition zones remained within the expected range when stored in the Radian® carousel. Additional graphs can be found in supplementary material (Fig. S1–S10).

**Table 2:** Stability of antibiotic disks when stored in the Radian® carousel expressed as ‘minimum amount of days; maximum amount of days’ found during the five repeats of the experiment

Antibiotic	Strain									
	*E. coli* ATCC® 25922™	*E. coli* ATCC® 35218™	*P. aeruginosa* ATCC® 27853™	*K. pneumoniae* ATCC® 700603™	*S. aureus* ATCC® 29213™	*S. aureus* NCTC® 12493™	*E. faecalis* ATCC® 29212™	*E. faecalis* ATCC® 51299™	*S. pneumoniae* ATCC® 49619™	*C. jejuni* ATCC® 33560™
Penicillin					0;3				7;7	
Oxacillin					7;7	7;7			7;7	
Ampicillin	4;7				0;1		4;7		4;7	
Amoxicillin-clavulanic acid	7;7	4;7								
Piperacillin-tazobactam	7;7	7;7	6;7	7;7						
Temocillin	7;7									
Cefadroxil	5;7									
Cefepime	5;7		6;7							
Cefoxitine					7;7					
Ceftazidime	7;7		7;7	7;7						
Ceftriaxone	5;7			5;7						
Cefuroxime	6;7									
Meropenem	6;7		5;7							
Ciprofloxacin	7;7		7;7		7;7		7;7		6;7	7;7
Moxifloxacin	7;7				6;7				7;7	
Aztreonam	4;7		7;7	7;7						
Amikacin	7;7		7;7							
Gentamicin	7;7		7;7		4;7		7;7	7;7		
Clindamycin					5;7				4;7	
Erythromycin					4;7				4;7	7;7
Fosfomycin	7;7									
Rifampin					5;7				6;7	
Tetracyclin					6;7				4;7	7;7
Linezolid					4;7		7;7		7;7	
Nitrofurantoin	7;7				7;7		7;7		5;7	
Vancomycin							7;7	7;7	5;7	
Cotrimoxazol	7;7				4;7		4;7		7;7	

#### *Escherichia coli* ATCC® 25922™ and 35218™

All antibiotic disks were stable for at least 4 days for both ATCC® strains. A slight downwards trend was observed for meropenem after 5 days in ATCC® 25922™. For amoxicillin-clavulanic acid, a downwards trend was noticed after 5 days in ATCC® 35218™.

#### *Klebsiella pneumoniae* ATCC® 700603™

All antibiotic disks were stable for at least 5 days.

#### *Pseudomonas aeruginosa* ATCC® 27853™

All antibiotic disks were stable for at least 5 days. A slightly downwards trend was observed for meropenem after 5 days.

#### *Staphylococcus aureus* ATCC® 29213™ and NCTC® 12493™

For ATCC® 29213™, disk diffusion zones for ampicillin and penicillin did not match the EUCAST criteria already on day 1 in all five runs, resulting in a stability of 0 days. During the first run erythromycin only had a stability of 2 days and tetracycline of 0 days. The following runs showed a minimum stability of 4 days for erythromycin and 6 days for tetracycline. Therefore, the first run was discarded. All other antibiotics were stable for at least 4 days. NCTC® 12493™ showed stability of 5 days for all investigated antibiotic disks.

#### *Enterococcus faecalis* ATCC® 29212™ and 51299™

All investigated antibiotic disks were stable for at least 4 days.

#### *Streptococcus pneumoniae* ATCC® 49619™

All investigated antibiotic disks were stable for at least 4 days.

#### *Campylobacter jejuni* ATCC® 33560™

All investigated antibiotic disks were stable for 7 days.

## Discussion

This study is the first to compare EUCAST standardized disk diffusion testing using SIRScan® (i2a) with the fully automated WASPLab® setup from Copan, including the Radian® in-Line Carousel. Additionally, the study investigates the thermolability of antibiotic disks when stored at room temperature for up to 7 days.

Until recently, disk diffusion AST was mainly a manual process with the option to use semi-automated reader instruments. The availability of automated liquid-based systems, such as VITEK® 2 (Biomérieux) and Phoenix™ (BD), and the amount of manual work have limited the use of disk diffusion in routine clinical microbiology laboratories. Dauwalder et al. described the automation of disk diffusion as being one of the last pieces missing for full microbiology laboratory automation [7]. Automating the system would expand the possibility of using disk diffusion as a reference method in many laboratories by reducing the workload. Despite its limitations, disk diffusion has several advantages over liquid-based systems. One significant advantage is its greater reliability in detecting heteroresistant profiles and certain carbapenemases. In 2019, Cherkaoui et al. performed an evaluation of the Copan WASPLab incorporating the BioRad expert system against SIRscan 2000, using 388 clinical strains. The inoculation method performed by WASPLab during this study mirrored the inoculum preparation utilized in the current study. Their findings demonstrated an accuracy that was equal or even better than the SIRscan 2000 [18]. However, the current study used the Radian® In-Line carousel for placement of the antimicrobial discs. Furthermore, we included a broader spectrum of resistant strains, such as CPE and multidrug resistant *P. aeruginosa*. In a subsequent study performed by Cherkaoui et al., the VITEK® 2 system was compared with automated disk diffusion using Radian® In-Line carrousel and found that the main cause of very major errors on the VITEK® 2 was due to heteroresistant populations of *P. aeruginosa* [3]. The advantage of disk diffusion is the visualization of these heteroresistant populations and antibiotic interactions.

An acceptable overall CA was obtained for all groups. Very major error rates were ≤ 5% for all antibiotics. Radian® was able to detect all CPE strains and all ESBL producing Enterobacterales strains using screening breakpoints provided by EUCAST for ESBL and CPE detections. This is in line with the results obtained by Cherkaoui et al. [3]. Every MBL producing *P. aeruginosa* was detected using CPE screening breakpoints provided by EUCAST. All VRE and MRSA strains were categorized correctly using EUCAST guidelines.

During the thermostability study, questionable results were obtained for *S. aureus* ATCC® 29213™ for ampicillin and penicillin disks. The disk diffusion inhibition zones were below the allowed range from day 1. This problem was addressed to the corresponding companies, and we chose not to include this in our study since the issue only occurred with this specific reference strain, and both antibiotics are not used to treat *S. aureus* infections and are not reported to the clinicians in routine. As of yet, the cause of this problem is still unidentified. Overall, thermostability of the antibiotic disks was verified for at least 4 days when stored at room temperature in the WASP Radian® carousel, facilitating the full automation of AST. While some manufacturers, such as MAST, provide longer stability periods for their antimicrobial discs, it is essential to validate stability within Radian® In-Line carousel. This validation is necessary as the relative humidity within the carousel may differ from that within a manufacturer-provided dispenser with desiccant.

Overall, automation of disk diffusion reduces the workload for laboratory technicians, as manual streaking of MH-plates, placement of antimicrobial disks, and measurement and interpretation of the inhibition zones is time consuming and require significant hands-on time. In contrast, minimal hands-on time is needed for loading the WASP and Radian® carousel, the inhibition zones are measured automatically and require sporadic manual adjustments.

A possible limitation of this study is the relatively small amount of isolates included. However, these isolates were selected to test the capability of the system. They include a challenge panel of bacterial strains selected by the Belgian national antimicrobial susceptibility testing committee [13]. This panel consists of 14 Gram-negative and 14 Gram-positive bacteria covering most important resistance  mechanisms and showing stable susceptibility results with both micro-dilution methods and disk-diffusion methods. In addition, we selected isolates from national quality control surveys with known resistance mechanisms. An additional potential limitation is the use of antimicrobial discs from two different manufacturers for disk diffusion by SirScan versus Radian® during this evaluation. As highlighted in a study from EUCAST, variations in the quality of disks exist between different manufacturers. However, in their 2017 evaluation, the discs from SirScan (i2a) and Oxoid (Thermo Fisher Scientific), used during this study, demonstrated comparable performance [19].

## Conclusion

In conclusion, this study demonstrates that the Radian® In-Line carousel is a reliable and cost-effective automated disk diffusion method. This method is also flexible and enables visualization of antibiotic interactions. The full automation of AST using disk diffusion provided by Copan may facilitate the implementation of this technique in routine clinical microbiology laboratories.

### Supplementary Information

Below is the link to the electronic supplementary material.

Supplementary file1 (DOCX 2201 KB)

## Data Availability

The data that support the findings of this study are available from the corresponding author upon reasonable request.
