# Supplementary material for: Evaluation of Automated Disk Diffusion Antimicrobial Susceptibility Testing Using Radian® In-Line Carousel
Source: Curr Microbiol. 2024 May 30;81(7):196. doi: 10.1007/s00284-024-03710-z (PMC11139706; doi:10.1007/s00284-024-03710-z)
Supplement: Supplementary file 1 — Supplementary file1 (DOCX 2201 KB) [file 284_2024_3710_MOESM1_ESM.docx]

**Validation of automated disk diffusion antimicrobial susceptibility testing using Radian^®^ in-line carousel**

***Supplemental material***

Kim Callebaut^1^, Anke Stoefs^1^, Kristof Emmerechts^1^, Kristof Vandoorslaer^1^, Ingrid Wybo^1^, Deborah De Geyter^1^, Thomas Demuyser^1,2^, Denis Piérard^1^ and Astrid Muyldermans^1^

^1^ *Department of Microbiology and Infection control, Vrije Universiteit Brussel (VUB), Universiteit Ziekenhuis Brussel (UZ Brussel), Laarbeeklaan 101, 1090 Brussels, Belgium.*

*^2^ AIMS lab, Center for Neurosciences, Faculty of Medicine and Pharmacy, Vrije Universiteit Brussel (VUB), Laarbeeklaan 103, 1090 Brussels, Belgium*

Corresponding author: Ingrid Wybo

[ingrid.wybo@uzbrussel.be](about:blank)

Tel +32 2 477 50 00 - Fax +32 2 477 50 15

Vrije Universiteit Brussel (VUB), Universitair Ziekenhuis Brussel (UZ Brussel)

Department of Microbiology and Infection Control

Laarbeeklaan 101, 1090 Brussels, Belgium


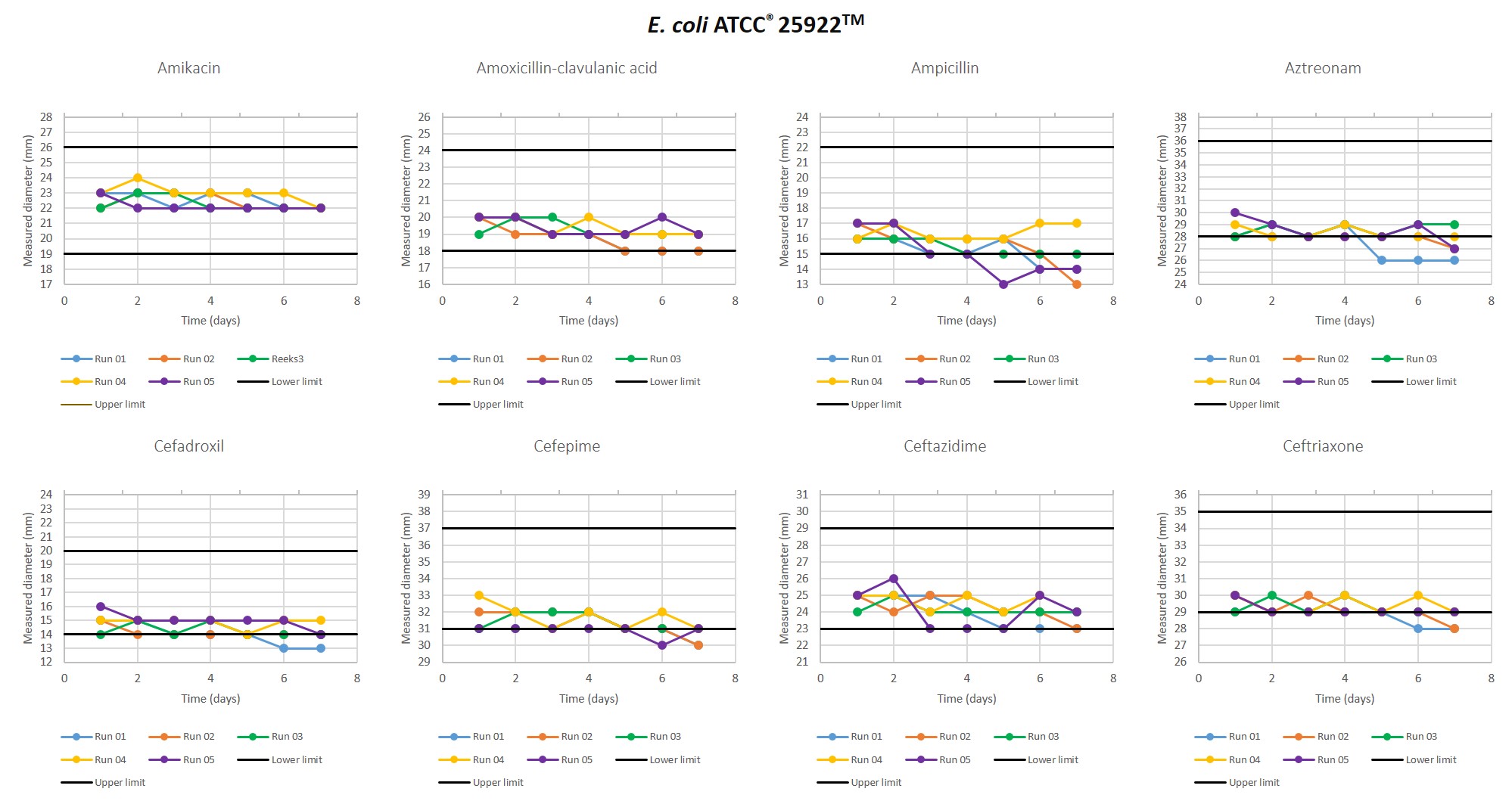

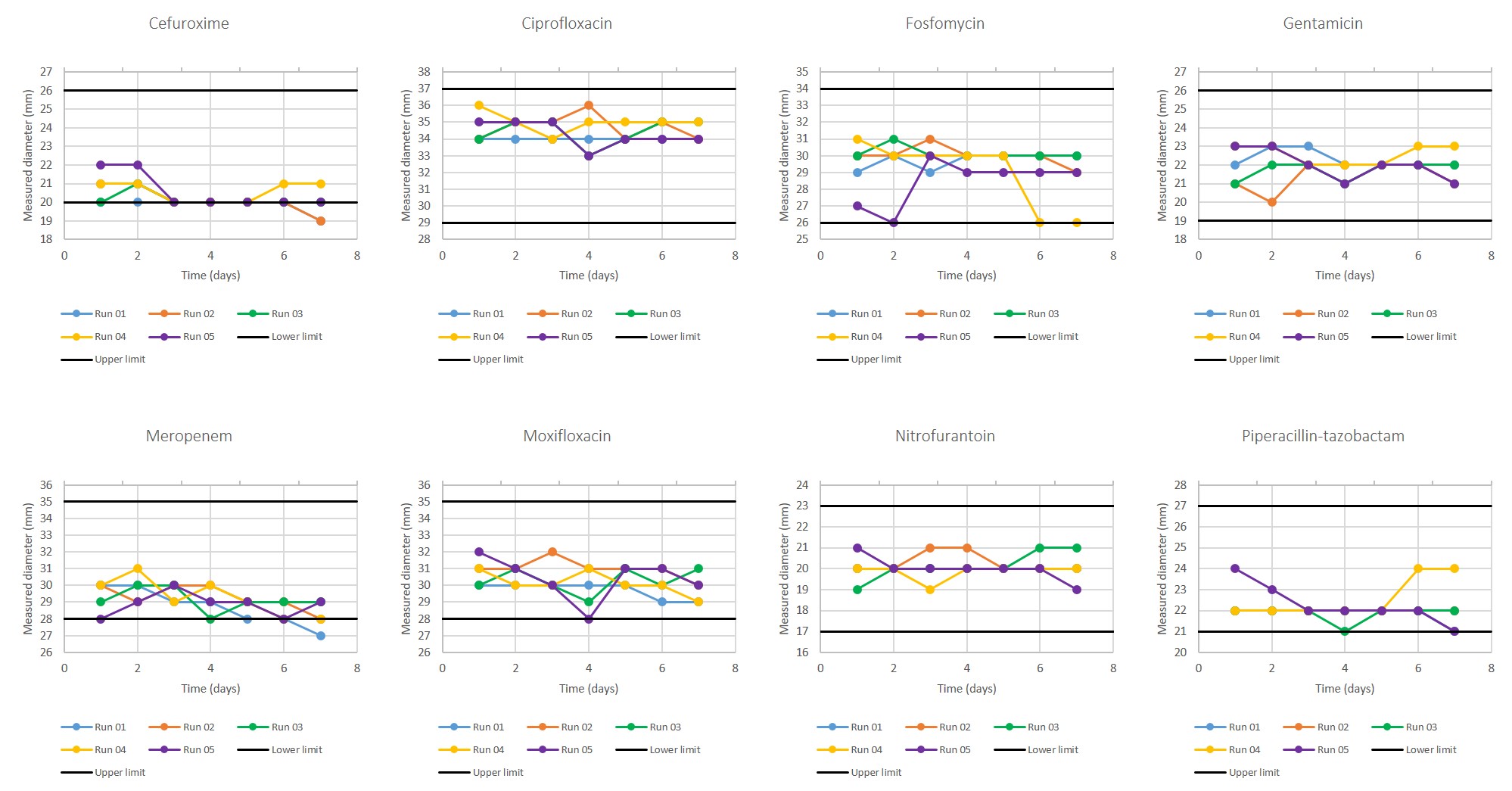

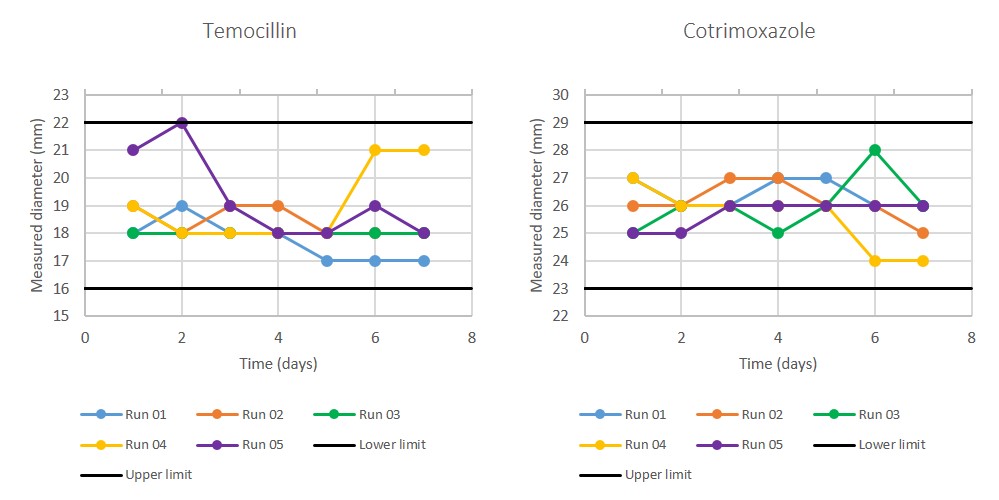


**Supplemental material, figure S1.** Stability of antibiotic disks when stored at room temperature in the Radian^TM^ carousel during seven days for ATCC^®^ 25922^TM^: *E. coli*


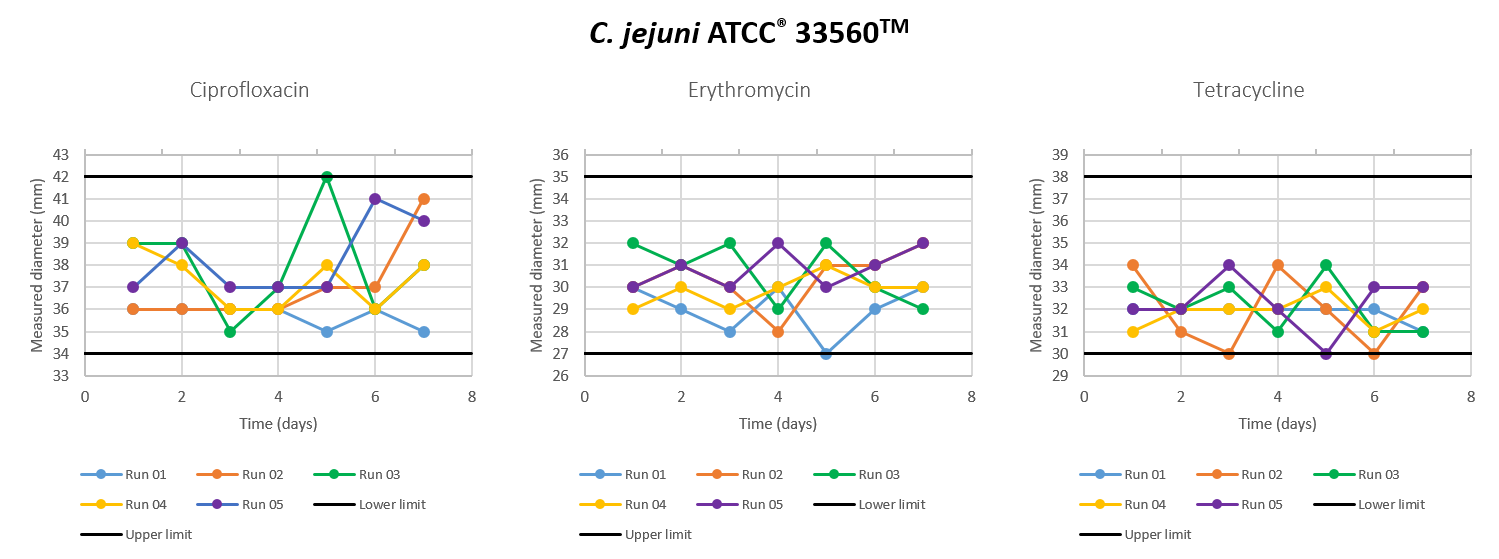


**Supplemental material, figure S2.** Stability of antibiotic disks when stored at room temperature in the Radian^TM^ carousel during seven days for ATCC^®^ 33560^TM^: *C. jejuni*


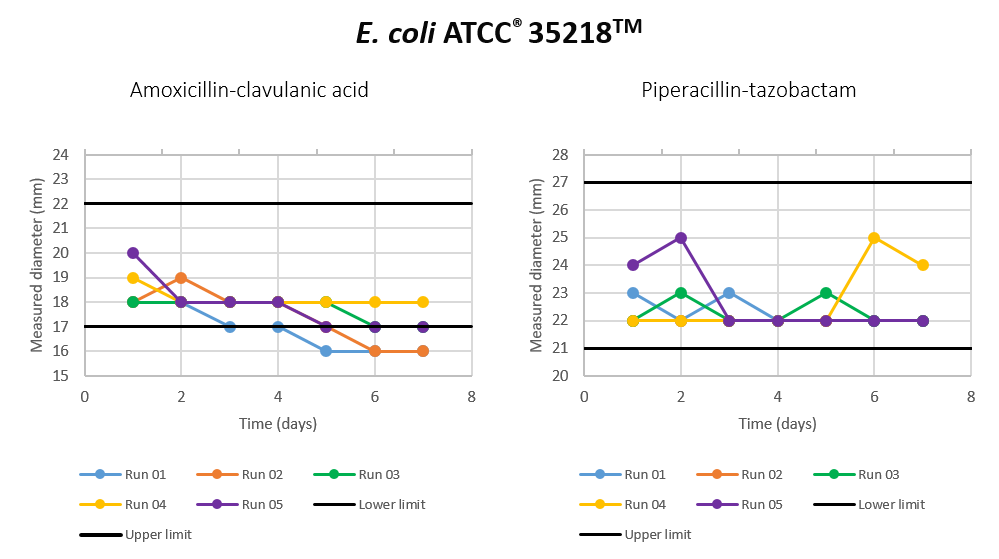


**Supplemental material, figure S3.** Stability of antibiotic disks when stored at room temperature in the Radian^TM^ carousel during seven days for ATCC^®^ 35218^TM^: *E. coli*


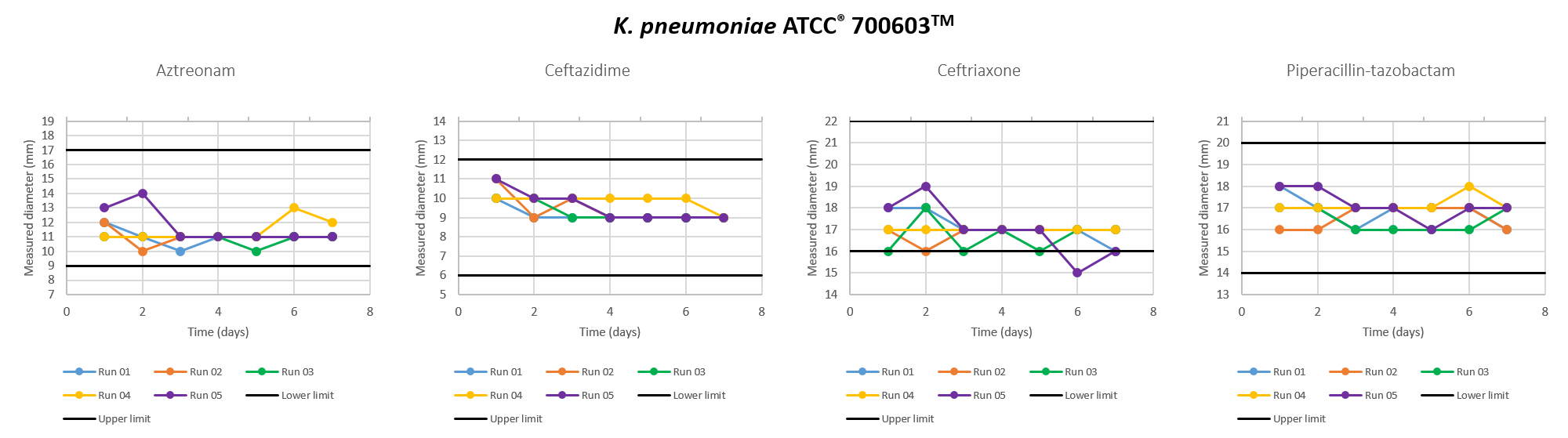


**Supplemental material, figure S4.** Stability of antibiotic disks when stored at room temperature in the Radian^TM^ carousel during seven days for ATCC^®^ 700603^TM^: *K. pneumoniae*


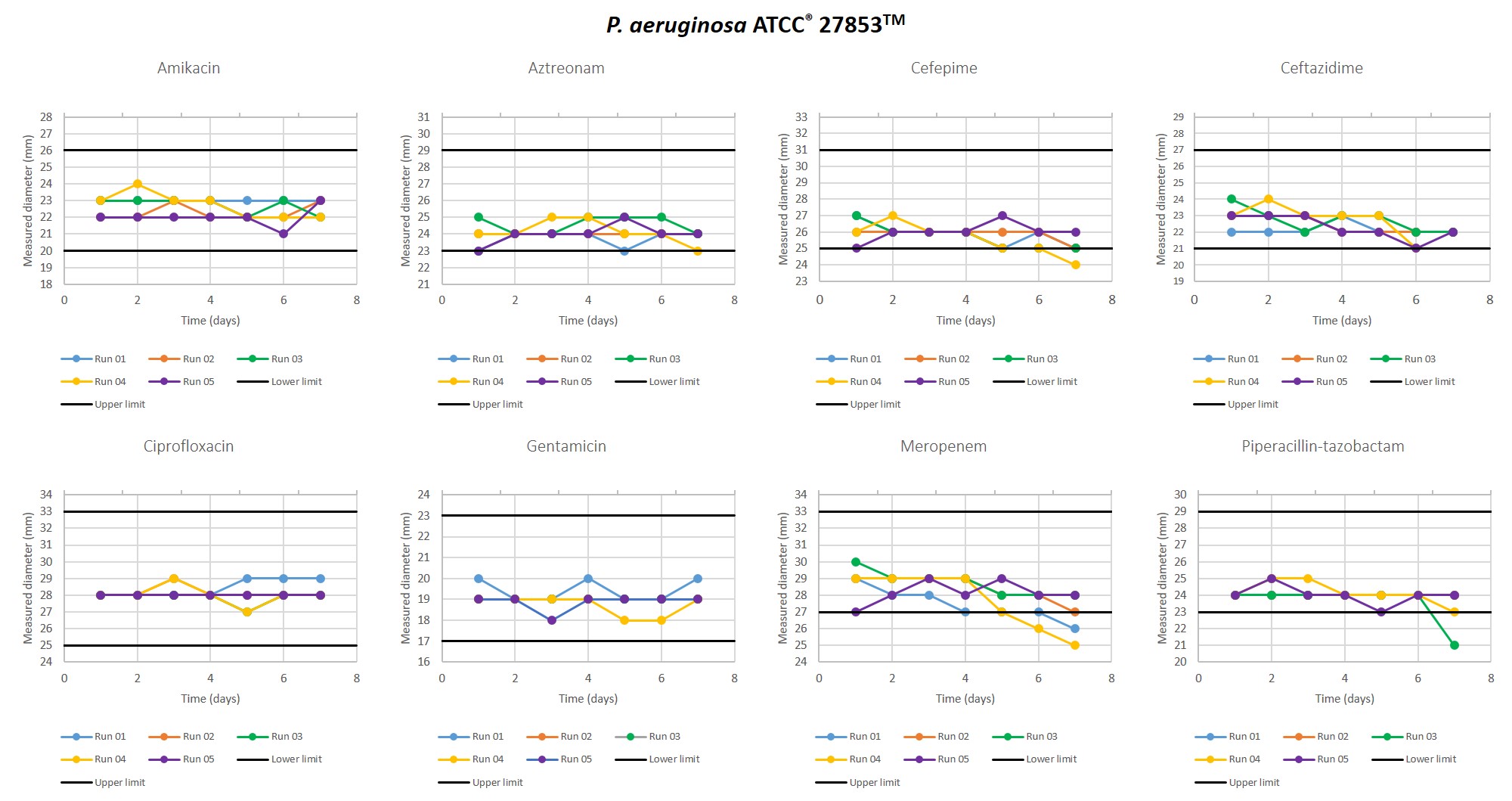


**Supplemental material, figure S5.** Stability of antibiotic disks when stored at room temperature in the Radian^TM^ carousel during seven days for ATCC^®^ 27853^TM^: *P. aeruginosa*


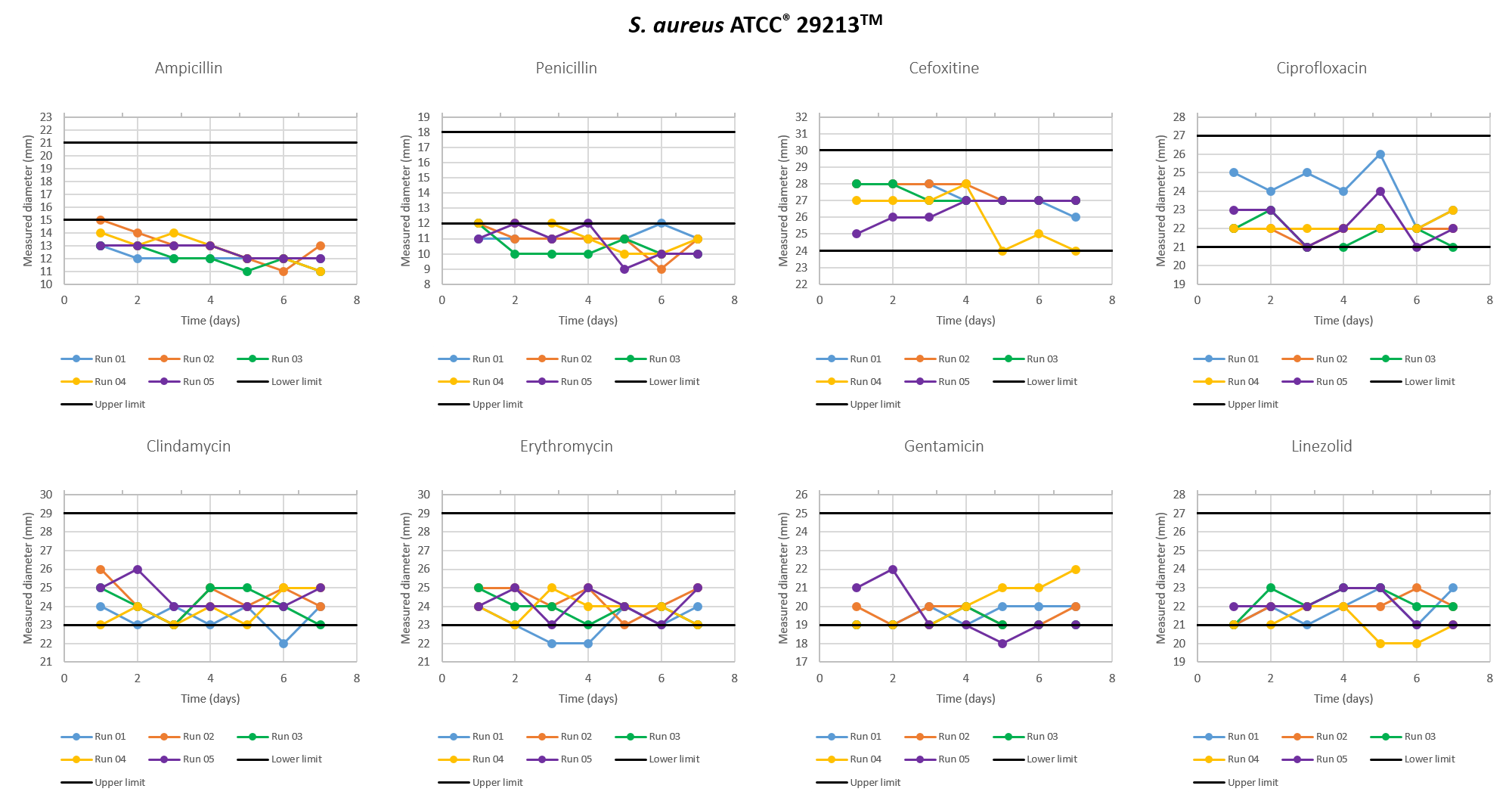

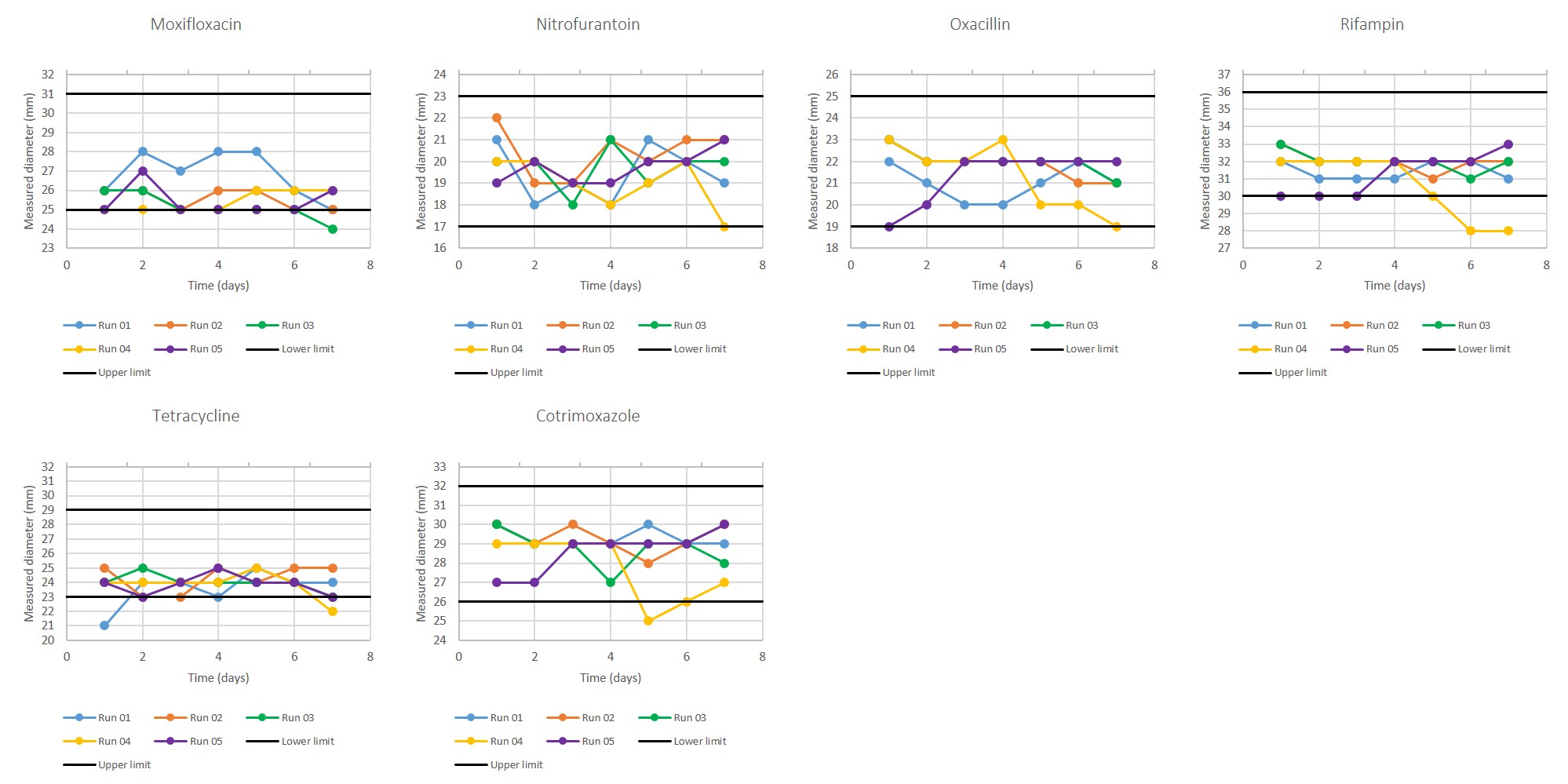


**Supplemental material, figure S6.** Stability of antibiotic disks when stored at room temperature in the Radian^TM^ carousel during seven days for ATCC^®^ 29213^TM^: *S. aureus*


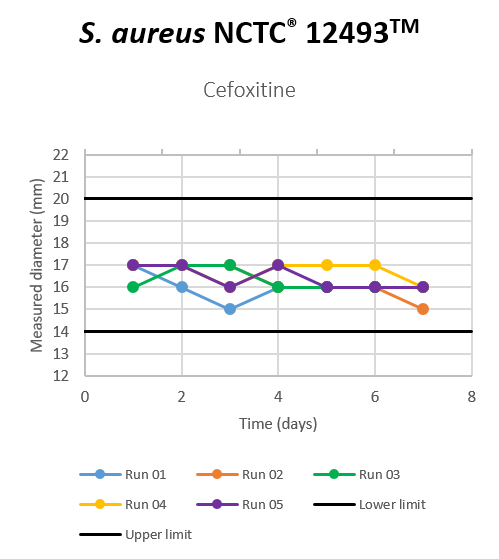


**Supplemental material, figure S7.** Stability of antibiotic disks when stored at room temperature in the Radian^TM^ carousel during seven days for NCTC^®^ 12493^TM^: *S. aureus*


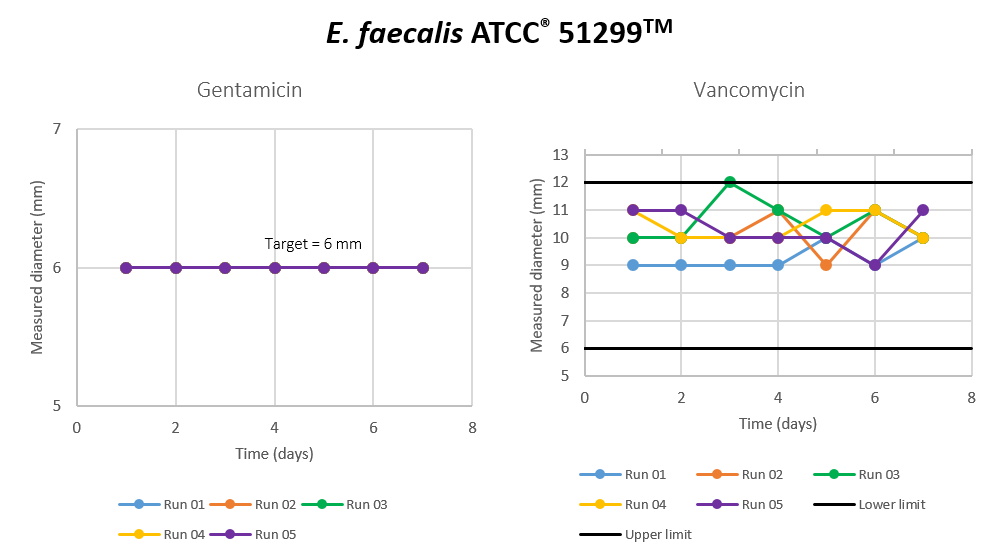


**Supplemental material, figure S8.** Stability of antibiotic disks when stored at room temperature in the Radian^TM^ carousel during seven days for ATCC^®^ 51299^TM^: *E. faecalis*


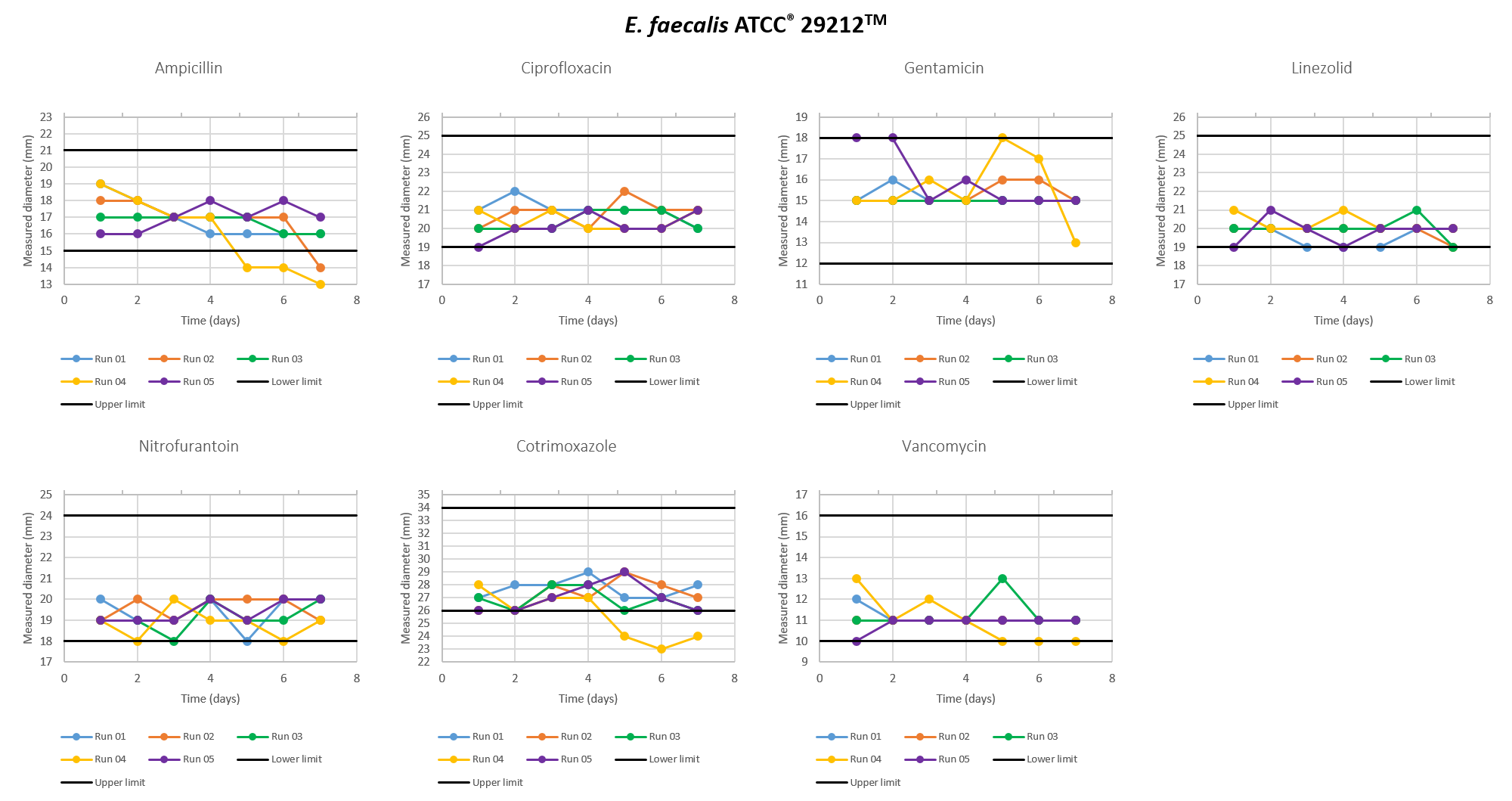


**Supplemental material, figure S9.** Stability of antibiotic disks when stored at room temperature in the Radian^TM^ carousel during seven days for ATCC^®^ 29212^TM^: *E. faecalis*


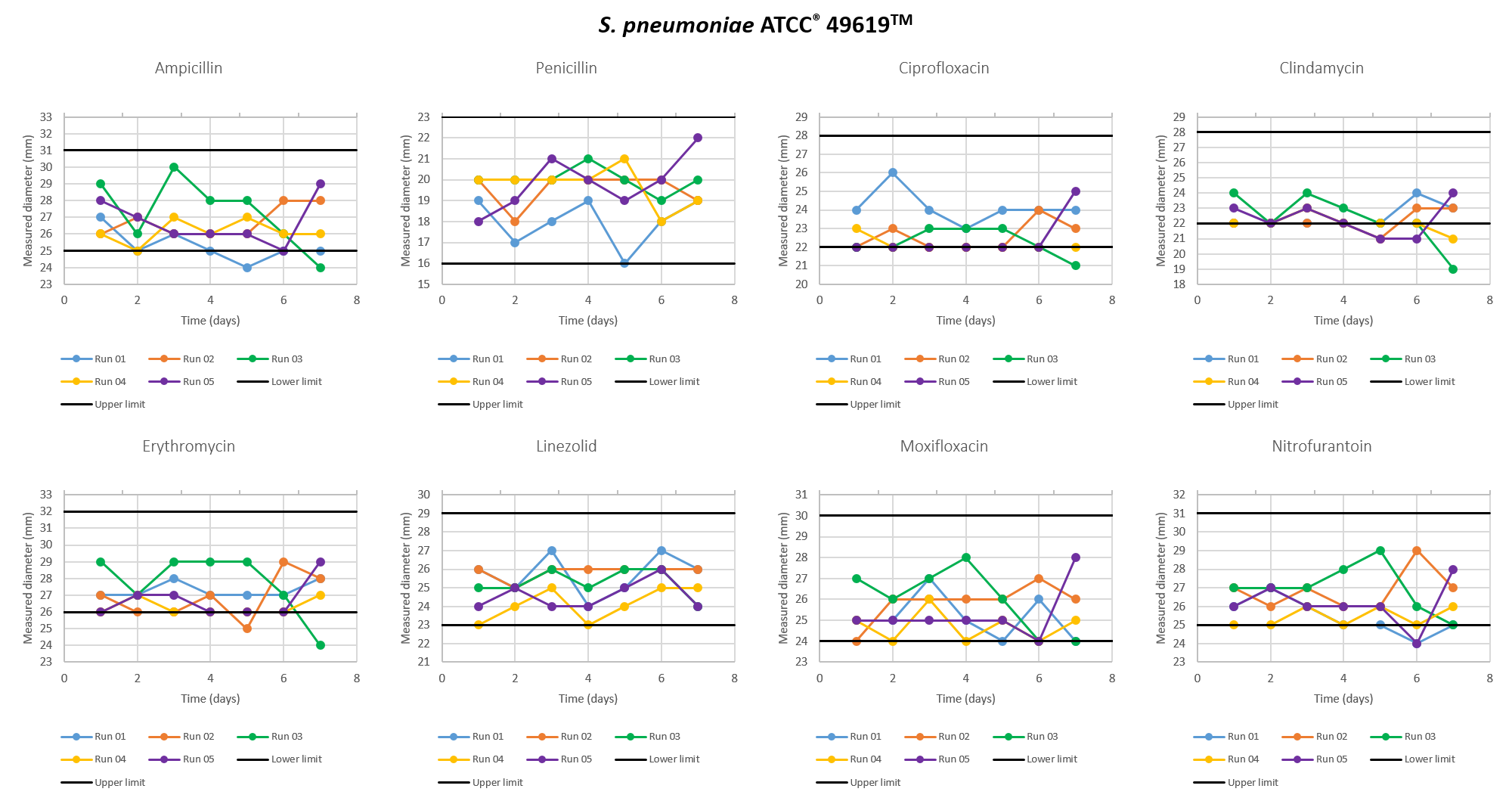

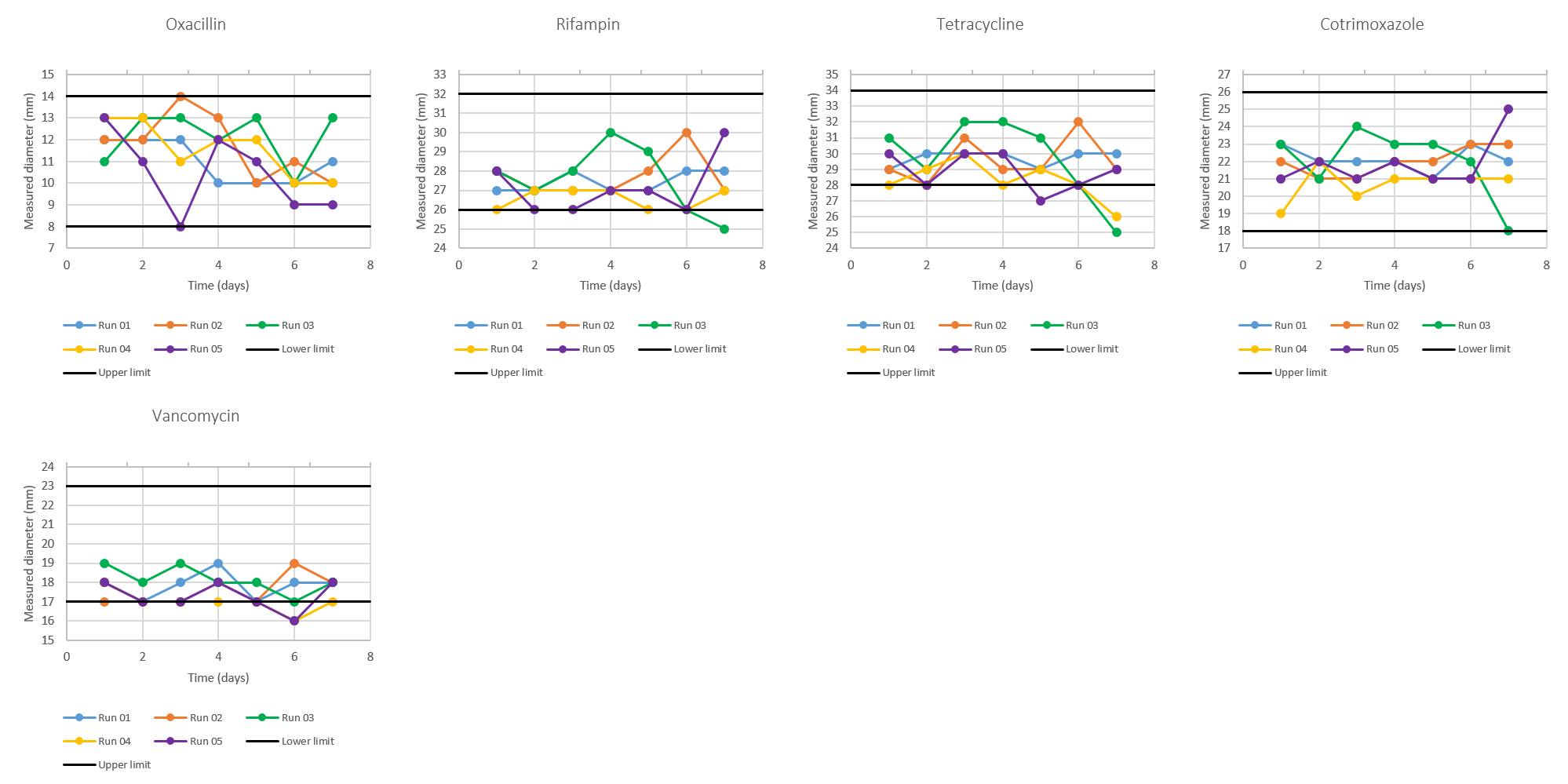


**Supplemental material, figure S10.** Stability of antibiotic disks when stored at room temperature in the Radian^TM^ carousel during seven days for ATCC^®^ 49619^TM^: *S. pneumoniae*
